# 双肺多发炎性假瘤1例

**DOI:** 10.3779/j.issn.1009-3419.2012.04.10

**Published:** 2012-04-20

**Authors:** 昕 李, 森 韦, 清华 周, 军 陈

**Affiliations:** 300052 天津，天津医科大学总医院肺部肿瘤外科，天津市肺癌研究所 Department of Lung Cancer Surgery, Tianjin Medical University General Hospital, Tianjin Lung Cancer Institute, Tianjin 300052, China

**Keywords:** 肺肿瘤, 炎性假瘤, 诊断, 治疗, Lung neoplasms, Inflammatory pseudotumor, Diagnosis, Treatment

## Abstract

炎性假瘤是一种肺部少见的良性肿瘤，其发病率仅占肺部肿瘤的0.7%，而双肺多发炎性假瘤更为罕见，在临床工作中常被误诊为转移瘤或播散性肺结核，国内外文献报道尚少。本文介绍临床工作中遇到的罕见双肺多发炎性假瘤1例，并初步探讨其诊断和治疗。

## Abstract (en)

Inflammatory pseudotumor was a kind of rare benign tumor of the lung, whose incidence was only about 0.7% in all kinds of tumors of the lung. Multiple inflammatory pseudotumor in both lungs were rare, which were often misdiagnosed to metastatic tumor or disseminated pulmonary tuberculosis. The literature reports were scarce too. This report introduces one case of multiple inflammatory pseudotumor in both lungs that we met in clinical works, and discusses the diagnosis and treatment of it.

## 1 临床资料

患者，女，60岁，因"咳嗽2月余，发现双肺结节2周"于2011年10月17日入院。患者2个月前受凉后出现咳嗽症状，干咳，无痰，无胸闷憋气，无发热，无胸痛，无恶心呕吐等不适。自服左氧氟沙星1周，症状无明显缓解，于当地医院行胸片检查提示肺部阴影，胸部CT提示双肺多发实性及亚实性结节，性质待定。患者为进一步诊治来我院就诊，以"双肺多发结节"入院。病程中精神和食欲佳，体重无明显变化。入院查体未发现明显异常，实验室检查未见异常。胸部强化CT+双肺结节三维重建(2011-10-19，图 1)示双肺多发结节，边缘毛糙，较大者直径约2 cm，部分与膈肌相连，首先考虑恶性肿瘤性病变。上腹部强化CT、全身骨扫描和头部增强核磁均未见明显异常。完善术前检查后于2011年10月20日在全麻下行电视胸腔镜下右肺结节切除活检。术中切除右肺上叶结节2个、中叶结节1个和下叶结节2个。病理报告为右肺结节5枚，边界清楚，镜下部分肺组织结构破坏，淋巴组织片状增生，混有大量组织细胞增生，其周边见机化性肺炎病变。免疫组化检测示CD20小片状阳性，PAX-5灶性弱阳性，CD3和CD68弥漫散在阳性，CD34血管阳性，CD23阴性(图 2)，考虑为肺部炎性假瘤。术后抗炎治疗5 d后复查胸部CT，提示双肺剩余结节大小无明显改变(2011-10-25，图 3)。患者病情好转出院，目前呼吸内科继续治疗及复查。

**1:**
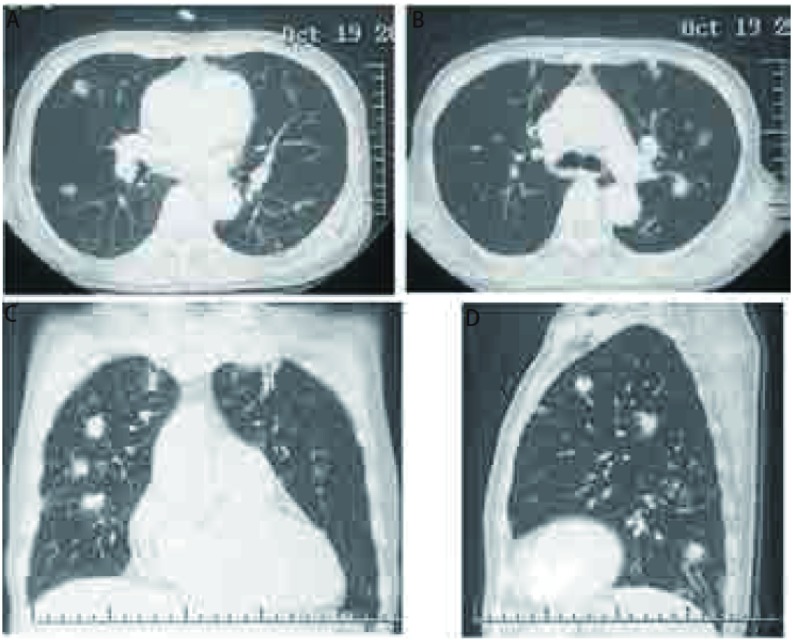
胸部CT检查。A，B：肺窗水平面显示双肺多发结节，边缘毛糙，较大者直径约2 cm；C：冠状面显示双肺多发结节；D：矢状面显示肺部多发结节。 CT scans of the chest. A, B: In lung window, horizontal, multiple nodules in both sides of the lung, crude border, the biggest one's diameter was about 2cm; C: In lung window, coronal, multiple nodules in both sides of the lung; D: In lung window, sagittal, multiple nodules in both sides of the lung.

**2:**
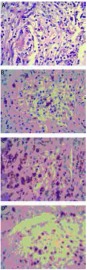
病理检查（×400）。A：HE染色显示肺部病变呈机化性肺炎改变伴片状淋巴组织增生；B：免疫组化显示CD20小片状阳性；C：免疫组化显示CD68弥漫散在阳性；D：免疫组化显示CD3弥漫散在阳性。 Pathological images (×400). A: HE staining of multiple nodules indicated the inflammatory change of pulmonary with lymphatic tissue proliferation; B: Immunohistochemical staining of primary tumor with antibodies to CD20 showed the positive staining. C: Immunohistochemical staining of primary tumor with antibodies to CD68 showed the positive staining; D: Immunohistochemical staining of primary tumor with antibodies to CD3 showed the positive staining.

**3:**
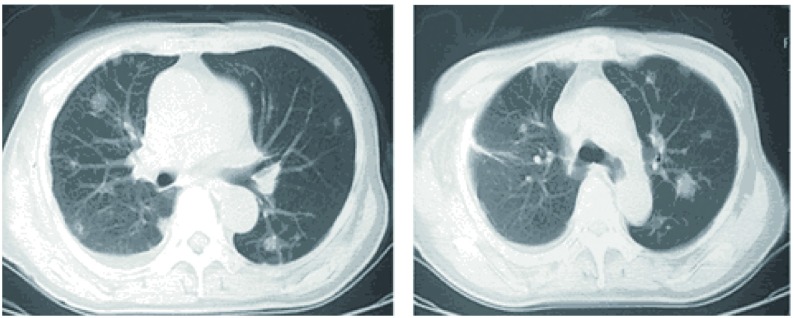
术后胸部CT检查示双肺剩余结节未见明显变化 CT scans of the chest show that the left nodules in both lungs has not change obviously

## 2 讨论

双肺弥漫性结节影是肺科在临床工作中常见的疾病影像学表现，但要明确诊断却比较困难，因为很多情况下双肺弥漫性结节影不具有特异性，单靠结节影的影像学特征不能确定病变类型^[1]^。高分辨CT+结节三维重建能显示肺部多发结节的分布特点、背景及内部结构，有助于肺弥漫性结节的诊断和鉴别诊断^[2]^，但最终诊断往往需要病理学确诊。需注意的是有时单个标本结果可能不满意，但不能因此而认为所有结节是同一病理性质，以免误诊，需要进行多点取材及多个标本完整切除活检。

肺炎性假瘤并非恶性肿瘤，根据其组织成分的不同曾被称为浆细胞肉芽肿、炎性肌纤维母细胞瘤、黄色瘤等。绝大部分甚至所有的炎性假瘤起源于机化性肺炎，由此逐渐演变为炎性假瘤。Kaitoukov等^[3]^认为某些病例中损害可能由炎症修复过程引起，也可能是肺部感染性疾病的一种特殊末期疾病。炎性假瘤临床并不多见，仅占肺肿瘤的0.7%，双肺多发炎性假瘤更为罕见，国内外报道尚少。

炎性假瘤在临床上可表现为发热、咳嗽、咳痰、咯血、胸痛或全身非特异症状，如贫血、体重下降、乏力等。肺多发炎性假瘤胸部CT可见多个圆形或卵圆形实性团块或结节，边缘整齐或不整齐，伴或不伴钙化，可有局部浸润，如累及纵隔、肺门、胸膜等，常误诊为转移性肺癌。有文献^[4]^报道，在最初接诊时被误认为肺转移瘤。术中大体形态可见肺多个灰白色结节，可有胸膜凹陷，结节直径0.2 cm-2.5 cm，也曾有报道最大直径为5 cm的炎性假瘤结节。手术是炎性假瘤的常用治疗手段^[5]^，但双肺多发炎性假瘤若勉强行根治性手术切除则对患者损伤过大，因此笔者认为此病例并不适合根治性手术，而采用小切口开胸或胸腔镜下单侧或双侧肺多个结节完整切除活检对于明确诊断是较为适合的方法，因开胸或胸腔镜下结节切除活检取得材料较经皮肺穿刺所取得的组织更完整，且多点取材代表性更强，因此有更高的阳性率及准确率，以免漏诊可能存在的恶性病变。

对于肺多发炎性假瘤的治疗，国外曾有报道^[5]^使用糖皮质激素治疗获得良好效果。而国内报道尚少，近10年来仅有2例关于糖皮质激素对于双肺多发炎性假瘤治疗有效的报道^[6, 7]^。因此，对于双肺多发的炎性假瘤、不能切除的和/或复发病例，其治疗方法还有待进一步研究探讨。
